# Etiologies of sperm oxidative stress

**Published:** 2016-04

**Authors:** Parvin Sabeti, Soheila Pourmasumi, Tahereh Rahiminia, Fatemeh Akyash, Ali Reza Talebi

**Affiliations:** *Research and Clinical Center for Infertility, Shahid Sadoughi University of Medical Sciences, Yazd, Iran. *

**Keywords:** *Oxidative stress*, *Sperm*, *Reactive oxygen species*, *Etiology*

## Abstract

Sperm is particularly susceptible to reactive oxygen species (ROS) during critical phases of spermiogenesis. However, the level of seminal ROS is restricted by seminal antioxidants which have beneficial effects on sperm parameters and developmental potentials. Mitochondria and sperm plasma membrane are two major sites of ROS generation in sperm cells. Besides, leukocytes including polymer phonuclear (PMN) leukocytes and macrophages produce broad category of molecules including oxygen free radicals, non-radical species and reactive nitrogen species. Physiological role of ROS increase the intracellular cAMP which then activate protein kinase in male reproductive system. This indicates that spermatozoa need small amounts of ROS to acquire the ability of nuclear maturation regulation and condensation to fertilize the oocyte. There is a long list of intrinsic and extrinsic factors which can induce oxidative stress to interact with lipids, proteins and DNA molecules. As a result, we have lipid peroxidation, DNA fragmentation, axonemal damage, denaturation of the enzymes, over generation of superoxide in the mitochondria, lower antioxidant activity and finally abnormal spermatogenesis. If oxidative stress is considered as one of the main cause of DNA damage in the germ cells, then there should be good reason for antioxidant therapy in these conditions.

## Introduction

Male fertility depends on spermatogenesis process which produces the large numbers of cell by the testis known as spermatozoa (1). Oxygen is important for aerobic metabolism of spermatogenic cells, but this molecule may have detrimental effects on cells via production of ROS. Significant positive correlation has reported between levels of reactive oxygen species (ROS) and percentage of spermatozoa with many kinds of abnormalities like, abnormal heads, acrosome abnormalities, mid piece anomalies, cytoplasmic droplets and tail defects (2). In fact, enhancement of ROS level in semen produced by abnormal spermatozoa can be a main cause of sub-fertility and even infertility. For example, teratozoospermic men who have a high level of ROS in their semen, show subfertility or even infertility (3, 4). ROS production rate is a main parameter in oxidative stress condition, which is the imbalance between ROS level and body’s antioxidant capacity (5-7).

Sperm, as well as critical phases of spermiogenesis are particularly susceptible to ROS induced damages for several reasons including; a) The period of sperm chromatin condensation is very susceptible, b) Sperm cells don’t have DNA repair mechanisms, c) Sperm membrane contain high levels of poly unsaturated fatty acids, d) Sperm themselves produce ROS, especially during passage through epididymis, e) Sperm possess low levels of cytoplasmic antioxidant enzymes (because most of the antioxidant enzymes are lost in spermiogenesis) and f) Sperm spend long periods as isolated cells in both male and female genital tracts (8-12). However, the level of seminal ROS is restricted by seminal antioxidants such as β-mercaptoethanol, protein, vitamin E, vitamin C, cysteamine, cycteine, taurin and hypotaurin (13-24). In fact, these compounds have beneficial effects on sperm parameters and developmental ability of embryos (25).

In this article, we reviewed the free radicals actions in male reproductive system and the effects of antioxidants on oxidative stress and fertility potential of spermatozoa. 

**Sources of ROS**

Different cell types in human ejaculate including seminal leukocytes and abnormal spermatozoa have been shown to be the main sources of ROS generation (26). It has been demonstrated that the morphologically abnormal spermatozoa are very active in ROS productions (27). Between different sperm anomalies, residual cytoplasm or cytoplasmic droplet may be considered as most important one in ROS production (28). Normally, these excessive cytoplasms are omitted by Sertoli cells during spermiogenesis (29, 30). However, the spermatozoa with this abnormality are immature and functionally retarded (31, 32). Studies have shown that ROS generation in cytoplasmic droplets is mediated by cytosolic enzyme, called “glucose-6-phosphate dehydrogenase “(G6PD), which induces high levels of ROS production during two pathways, I) nicotinamid adenine dinucleotide phosphate (NADPH) located in sperm plasma membrane and II) NADPH- dependent oxido-reductase known as diphorase located in middle piece at mitochondrial respiratory level (33-37). In fact, mitochondria and sperm plasma membrane are two major sites of ROS generation in sperm cells (38).

There are large numbers of mitochondria in middle piece of sperm cells which supply energy for their motility. Mitochondrial dysfunction is related to ROS production and ROS can affect mitochondrial integrity in spermatozoa, that is a mutual cycle; ROS causing injury to mitochondrial membrane and injured mitochondrial membrane causing an enhancement in ROS production (35, 39). On the other hand, in a recent study, it was shown that NADPH oxidase 5 (NOX5) of spermatozoa plays an important role in ROS production (40). 

Another source of ROS in seminal fluid is Peroxides-positive leukocytes including polymorphonuclear (PMN) leukocytes (50-60% of all seminal leukocytes) and macrophage (40-50% of the rest) (41, 42). It has shown that activated leukocytes in response to different inducers like infection and inflammation can produce up to 100 fold higher levels of ROS compared with non-activated leukocytes (8, 43). The World Health Organization (WHO) defines “Leukocytospermia” as the increased leukocytes infiltration in semen with concentration more than 1×10^6^/ml (44).

**Types of ROS**

Reactive oxygen species represent a broad category of molecules including: a) Oxygen free radicals, such as superoxide anion (O_2_), hydroxyl radical (OH) and hyperoxyl radical (HOO). b) Non radical species, such as hypochlorous acid (HOCl) and hydrogen peroxide (H_2_O_2_). c) Reactive nitrogen species and free nitrogen radicals such as nitroxylion, nitrous oxide, peroxynitrite, etc. (1, 45, 46).

**Physiological role of ROS in male reproductive system**

Aitken and his group were the pioneers of research and study in the field of physiological role of ROS, in male reproductive system (47). Several study, have indicated that spermatozoa needs small amounts of ROS, to acquire the ability of fertilizing the oocytes (48-51). It is also demonstrated that spermatozoa need a small amounts of ROS, for capacitation, hyperactivation, motility, acrosome reaction and fertilization (52, 53). In fact, ROS, along with several factors in spermatozoa, increase intracellular cAMP which then activates protein Kinase A. These changes, in turn, increase tyrosine phosphorylation, that is the major driving force for capacitation (54). 

In addition, increase in cAMP levels, leading to an increase in sperm motility or hyper activation. Also, as a result of capacitation, acrosome membrane becomes unstable, then several hydrolytic enzymes like acrosin are released during acrosomal reaction and allowing sperm to binds oocyte (55, 56). It has been suggested that, ROS, take part in the regulation of nuclear maturation in spermatozoa. According to this, these reactive agents produce the lipid peroxides, and they probably provide a substrate for GPX4 (Phospholipid Hydroperoxide Glutathione Peroxidase), and so cause the oxidation of nuclear proteins and they facilitate nuclear condensation (57). In fact GPX4 is able to use thiols in nuclear protein, as an alternative reductant to glutathione (58).

**Etiologies of oxidative stress**

Although, there is a long list of intrinsic and extrinsic factors which can induce oxidative stress, but the main generally accepted etiologies are the followings:

**Alcohol consumption**

Several studies have shown that alcohol consumption, can increase the abnormalities in nucleous and plasma membrane of spermatozoa (59, 60). In an experimental study, it was shown that ethanol consumption increases the sperm cells percentage with chromatin abnormalities (61). In alcohol metabolism pathway, NADH and acetaldehyde are produced which, NADH increases the activity of respiratory chain in mitochondria and acetaldehyde interacts with lipids and proteins to produce ROS (62, 63).

**Cigarette smoking**

Cigarette smoking can decrease the sperm motility, number of normal sperm and produce reactive oxygen species by lipid peroxidation (64-67). In addition, it can decrease the antioxidants level , for example: vitamine E, vitamine C and increase in ROS level of seminal plasma (61, 68-71). Also, cigarette smoking can cause the inflammatory reaction and increase in leukocytes number in testicles (72, 73). The other changes that have been observed in smokers are including DNA fragmentation, axonemal damage, and reducing the sperm number (74-77).

**Varicocele**

Several studies have shown, that the oxidative stress in varicocele men could be the result of both increase in the level of ROS, and decrease in the total antioxidant capacity (78-80). In fact, high levels of ROS, and low levels of total antioxidant capacity, lead to impairment of cell membrane structure and DNA integrity of spermatozoa (30, 81). Talebi *et al* indicate that the varicocele patients have more spermatozoa with abnormal chromatin condensation than fertile controls (82). In addition, Ha *et al* showed that ROS may cause damage to blood-testis barrier in varicocele patients (83). Other studies have shown the presence of NO, in spermatic vein of varicocele men. NO, is a lipophilic molecule and it has cytotoxic effects on adjacent sperm cells. In addition, NO and superoxide which released by monocytes form peroxynitrite cause more spermatozoa damage (84, 85). Fisher *et al* stated that, sperm cells of varicocele patients have high cytoplasmic droplets which produce the high levels of ROS (86).

**Obesity**

Anthony *et al* demonstrated that ROS overproduction, and abnormal hormonal regulation in obese men lead to suboptimal semen quality (87). It is suggested that dysregulation of adipocytokine and ROS generation, are the causes of oxidative stress in these patients (88). Over production of ROS, could be due to increase the metabolic rates, and maintain hemostasis in obese men. Besides, increase in ROS production and temperature, in testicles, may denature the enzymes, implicated in spermatogenesis. Hjollund *et al* stated that, increasing the temperature of scrotal skin was associated with decrease in sperm concentration (89). Another study, indicate the negative correlation between sperm density and total count (90).

**Diabetes**

For the first time, Baynes linked the diabetes mellitus to ROS (91). In diabetic patients, we have the increase levels of ROS, and impairment of antioxidant defense capacity (92). However, oxidative stress which is related to hyperglycemia is due to over generation of superoxide in mitochondria (93, 94). Agbaje *et al* stated that, the level of sperm DNA fragmentation in diabetic men is higher than normal men (95). On the other hand, there are some data indicating the effects of experimentally-induced diabetes on sperm chromatin quality and DNA integrity (96-99).

**Physical exercise**

An interesting issue is that too much exercises, due to muscle aerobic metabolism, produce large amounts of ROS, which lead to oxidative stress (100). Manna *et al* observed that the rates of exercises are related to reduction in sperm motility and count which may be the result of testicular oxidative stress (101).

**Psychological stress**

Several studies have indicated that, psychological stresses increase the level of ROS in seminal plasma and decrease the antioxidant protection capacities which in turn lead to reduction in sperm quality. It is suggested that these negative effects of psychological stresses may be related to central destruction of gonadotropin drive (102-104).

**Aging**

Desai *et al* stated that, ROS production has a central role in age-related reduction of male fertility by affecting on aging biomarkers (105). In addition, several studies have shown, increasing sperm DNA damage with age in fertile and infertile men (106-108). An experimental study revealed that spermatozoa from older animals produce more free radicals compared with younger ones and the former have lower antioxidant activity, too (109, 110).

**Environment factors**

Environment pollutants may be considered as one of the main source of ROS production. De Rosa *et al*, indicated that NO and lead can diminish the seminal quality, and motor vehicles by releasing NO, have a negative effect on male fertility (111). It is shown that Pb can decrease sperm normal morphology, count and viability (112). Also, it was shown that, butyl benzyl phalate has toxic effect on testis and reduces the testosterone level in serum (113, 114). Lee *et al* in a study on rats, indicated that oral administration of phalate esters, increased the ROS production and reduce the level of antioxidant in testis and finally it causes disturbances in spermatogenesis (115).

Electro-magnetic waves from cell phones, by ROS production, have deleterious effect on sperm. In one study, the semen samples were exposed to radiofrequency electromagnetic waves and a significant reduction in sperm motility, viability, and an increase in the level of ROS, with reduction in ROS-TAC score were found (116-118).

**Infections**

ROS has endogenous and exogenous sources. The endogenous ROS produces by immature sperm and leukocytes of semen (50). The male reproductive ducts infections and inflammatory reactions can be considered as exogenous sources (119). A few studies demonstrated the elevation of ROS concentration in infectious diseases. Mazilli *et al* demonstrated that, in patients with sperm culture-positive for aerobic bacteria, the superoxide anion production was high (120). Also, a high ROS level in chronic non-bacterial inflammation was seen (121).

The major sources of seminal ROS are polymorphonuclear leukocytes. In fact, the bacterial products and cytokines, can increase the ROS generation in these cells (122, 123). In a variety of human infectious, ROS, are produced generally by viruses besides bacteria and parasites (124). When a virus enters the cell, it impairs the cellular functions and it leads to an inequality in ROS system (125). It has been shown that urinary tract infections cause an elevation in ROS level in sperm fraction after percoll separation (126). There is a strong relation between inflammation of male genital system and infertility (127, 128). Actually, ROS that are produced in testis infection and epididymis are dangerous for sperm, because, antioxidant protection in sperm is low and ROS can affect these cells in a long period (129, 130).

**Antioxidant and male fertility**

There is a balance between ROS production and antioxidant activity in male reproductive system in normal conditions. But overproduction of ROS in semen can affect sperm or seminal plasma antioxidant defense mechanisms and cause oxidative stress (50, 131, 132). The body has developed antioxidant defense system by scavenging and minimizing the formation of oxygen derived radicals to protect itself from oxidative damage (133, 134). Although, ROS have both physiological and pathological function, to keep this level in confident range, the human body has a system against ROS (135, 136). In fact, when the free radicals level increases pathologically, antioxidant activity activated to prevent ROS oxidative damage (137).

Seminal plasma has endogenous antioxidant for protecting spermatozoa from oxidative damage (138, 139). These antioxidants are divided to enzymatic and non-enzymatic antioxidant and male reproductive system has both antioxidant (1). Enzymatic antioxidant contains superoxide dismutase, catalase, and peroxidase that catalytically remove reactive oxygen species from biological systems. Sperm themselves predominantly process this enzymatic antioxidant (140). 

Total seminal antioxidant activity is also supplemented by non-enzymatic antioxidants in semen. Non-enzymatic antioxidants in semen are usually present in the form of vitamin C, vitamin E, beta carotenes, carotenoids, flavonoids and metal binding proteins such as albumin, ferritin, and myoglobin, that act as antioxidant by inactivating pro-oxidant transition metal ions (141-143). Seminal plasma has the main antioxidant role, defending the spermatozoa from the ROS produced by the immature sperm cells and leukocytes through non-enzymatic scavengers existing in semen (144, 145). However, the spermatozoa have minimum antioxidant enzymes. In addition, antioxidant enzymes in sperm cannot prevent tail and acrosome membranes from lipid peroxidation. In other word, the sperm cells also need extra antioxidant defense system (146).

It is generally accepted that antioxidant therapy can improve the sperm quality and male fertility. Vitamin C and its antioxidant property for improvement of sperm parameters is confirmed by several studies support the positive effects of vitamin C on different sperm parameters (147-149). It is shown that the addition of vitamin C and vitamin E to the sperm of normozoospermic and asthenozoospermic men reduce ROS induced DNA damage (16, 150). Treatment by vitamin E for 6 months can reduce the lipid peroxidation of spermatozoa and may increase the pregnancy rate in asthenozoospermic cases (151). 

Additionally, coadministration of vitamin E and selenium increases the sperm motility in infertile men (152, 153). It is also demonstrated that the administration of zinc, vitamin C and vitamin E in asthenozoospermic patients reduces oxidative stress, apoptosis and sperm DNA fragmentation index (154-156). Many studies have indicated the improvement in sperm count and motility of asthenozoospermic and oligospermic patient after carnitine intakes (157-162). Alone or co-administration of zinc and folic acid increases the sperm count in infertile men but not in fertiles (163, 164).

## Conclusion

In normal condition male reproductive system has a balance between ROS production and antioxidant activity. But, overproduction of ROS in semen can affect sperm or seminal plasma antioxidant defense mechanisms and cause oxidative stress. It is approved that oxidative stress elevates sperm chromatin/ DNA damage. A variety of several etiological factors including intratesticular, post-testicular, and external factors, such as alcohol, cigarette smoking, varicocele, diabetes and etc. have been correlated with increased levels of ROS and sperm DNA damage, and in turn, can affect the potential of male fertility. It is generally accepted that antioxidant therapy can improve the sperm quality and male fertility by reducing oxidative stresses.

## Conflict of interest

All investigators disclose no conflict of interest in this study.
